# A newly discovered variant of a hantavirus in Apodemus peninsulae, far Eastern Russia.

**DOI:** 10.3201/eid0705.017530

**Published:** 2001

**Authors:** L Yashina, V Mishin, N Zdanovskaya, C Schmaljohn, L Ivanov


tional PRNT results from 26 sera, of which 18 (69%) were positive, yields a 3.9% positivity rate.

In addition to our study, with crude seroprevalence rates ranging from 3.9% to 10.1%, another recent study demonstrated JCV antibodies in 2.9% to 13.3% of ill persons in Massachusetts (Tonry J et al., unpub. data). Although the screening results of our first serosurvey (10.1% positive) differed widely from those of the second serosurvey (3.9% positive), even the lower rate indicates substantial levels of human infection in Connecticut.

This report suggests that JCV infection is fairly frequent in Connecticut and that illness may occur, as corroborated by data from neighboring Massachusetts (Tonry J et al., unpub. data) and unpublished laboratory findings from the Connecticut State Public Health Laboratory. The interest in arboviral disease will continue unabated, spurred by the continued occurrence of WNV, and systematic testing for JCV infection may be timely, at least throughout the northeastern United States.

**Donald Mayo,* Nick Karabatsos,† Frank J. Scarano,‡ Timothy Brennan,* Daniel Buck,§ Terry Fiorentino,§ John Mennone,§ and San Tran§**

*Connecticut Department of Public Health Laboratory, Hartford, Connecticut, USA; †CDC Division of Vector-Borne Infectious Diseases, Fort Collins, Colorado, USA; ‡University of Massachusetts Dartmouth, Dartmouth, Massachusetts, USA; and §Yale University Department of Epidemiology and Public Health, New Haven, Connecticut, USA

### References

1. Grimstad PR. California group virus disease. In: Monath TP, editor. Arboviruses Vol. II. Boca Raton (FL): CRC Press 1988. p. 99-136.
2. Centers for Disease Control and Prevention. 2000 update: West Nile virus activity. MMWR Morb Mortal Wkly Rep 2000; 49:1044-7.
3. Zamparo JM, Andreadis TG, Shope RE, Tirrell SJ. Serologic evidence of Jamestown Canyon virus infection in white-tailed deer populations from Connecticut. J Wildl Dis 1997;33:623-7.
4. Sprana HE, Main AJ, Wallis RD. Jamestown Canyon virus in Connecticut. Mosq News 1978;38:392-5.

### A Newly Discovered Variant of a Hantavirus in *Apodemus peninsulae*, Far Eastern Russia

**To the Editor:** Hemorrhagic fever with renal syndrome (HFRS) is caused by *Hantaan virus* (HTNV) or *Seoul virus* (SEO) in Asia and *Puumala virus* (PUUV) or *Dobrava virus* (DOBV) in Europe (1). Each of these hantaviruses is predominantly associated with a single rodent species as its primary natural reservoir: HTNV with the striped field mouse *Apodemus agrarius*, SEO with *Rattus norvegicus*, PUUV with the bank vole *Clethrionomys glareolus*, and DOBV with the yellow-necked mouse *Apodemus flavicollis*. An additional rodent reservoir of DOBV, *A. agrarius*, was reported recently (2).

The first HFRS cases (then called "hemorrhagic nephroso-nephritis") were clinically described in the Amur River basin during the 1920s by Russian scientists (3). Serologic studies suggest that numerous hantaviruses are present in humans and rodents in the far east of Asian Russia (4-5). Serologic evidence of hantavirus infection in *A. agrarius, A. peninsulae* (Korean field mouse), *R. norvegicus, Cl. rufocanus, Cl. rutilus,* and *Microtus fortis* has been reported (5). Only *Khabarovsk virus* (KBR), isolated from *M. fortis,* has been characterized in detail, and no association with human disease was established (6).

To genetically characterize hantaviruses in *A. peninsulae,* we studied samples from rodents captured in July and August 1998 in the same region of the forest near Khabarovsk. Lung-tissue samples were screened by enzyme-linked immunosorbent assay for HTNV/SEO/PUUV-related antigen. Samples from four hantavirus-positive rodents were tested by reverse transcription and nested polymerase chain reaction (PCR). Four M-segment PCR products (nt 2639-3000) and two S-segment PCR products (nt 592-945) were produced and directly sequenced (GenBank accession numbers AF332569-AF332573). All sequences were closely related to each other, with nucleotide diversity between strains not exceeding 0.6% for M segments and 1.3% for S segments. Comparative analysis of the M segments showed that hantaviral nucleotide sequences from *A. peninsulae* were very similar to those we identified earlier in HFRS patients (diverging 3.1% to 6.6%), which we term the Amur genotype of HTNV (7). The S-segment sequences of the AMR genotype from human patients were not available for comparison. The nucleotide sequence (the M and S segments, respectively) of the hantavirus detected in *A. peninsulae* diverged substantially from those of other hantaviruses (15% and 19% for HTNV, 21% to 28% for SEO, 22% and 29% for DOBV, 38% and 39% for PUUV, and 36% and 37% for KBR).

Neighbor-joining phylogenetic analysis based on partial sequences of the S segment indicated that the hantaviral sequences from *A. peninsulae* form a separate lineage on the phylogenetic tree, and together with HTNV virus strain 76-118, which originates from *A. agrarius,* constitute a well-supported group. A phylogenetic tree based on partial M segment sequences placed all hantavirus strains originating from *A. peninsulae* or from HFRS patients apart from all HTNV sequences recovered from *A. agrarius* (strain 76-118) and HFRS patients from Korea (strains HoJo, Lee). The taxonomic placement of this hantavirus (Amur genotype) as a distinct hantavirus or a distinct genetic lineage of HTNV remains to be determined. In addition, the finding of distinct DOBV genetic lineages in *A. flavicollis* and *A. agrarius* raises the same question of whether the two DOBV variants represent distinct hantaviruses (2).

*A. peninsulae* is widely distributed throughout eastern Asia, from Altai and south Siberia to the Russian far east, northeastern and eastern parts of China, and Korea. A survey of hantavirus antigens in rodent populations in the far east of Russia demonstrated the presence of HTNV-like antigen in 8% to 16% of *A. peninsulae* (5). Whether pathogenic AMR genotype of virus exists in *A. peninsulae* throughout far eastern Asia, from Russia to China and Korea, requires further study. Comparing hantaviral genome sequences available from GenBank shows that the M segment nucleotide sequence recovered from an HFRS patient from China (strain H8205, GenBank accession number AB030232) was very similar to the AMR genotype from *A. peninsulae* (94% to

96% identity), suggesting that this hantavirus is also present in *A. peninsulae* in China.

In earlier studies, we found that sera from patients infected by the AMR genotype of hantavirus showed extensive cross-reactivity with HTNV and SEO antigens in immunofluorescent antibody tests (7). Consequently, many HFRS cases previously thought to have been caused by HTNV or SEO may instead have been caused by infection with the hantavirus described here.

Our data represent the first genetic evidence for the AMR genotype of HTNV in *A. peninsulae* and suggest that this rodent species may be a natural reservoir for this pathogenic hantavirus.

This work was supported by the Ministry of Sciences of the Russian Federation and the National Academy of Sciences of the United States through the International Science and Technology Center, grant # 805.

**Lyudmila Yashina,* Vasiliy Mishin,* Nina Zdanovskaya,† Connie Schmaljohn,‡ and Leonid Ivanov†**

*State Research Center of Virology and Biotechnology “Vector,” Koltsovo, Novosibirsk, Russia; †Khabarovsk Antiplaque Station, Khabarovsk, Russia; and ‡U.S. Army Medical Research Institute of Infectious Diseases, Fort Detrick, Frederick, Maryland, USA

### References

1. Schmaljohn C, Hjelle B. Hantaviruses: a global disease problem. Emerg Infect Dis 1997;3:95-104.
2. Avsic-Zupanc T, Nemirov K, Petrovec M, Trilar T, Poljak M, Vaheri A, et al. Genetic analysis of wild-type Dobrava hantavirus in Slovenia: co-existence of two distinct genetic lineages within the same natural focus. J Gen Virol 2000;81:1747-55.
3. Casals J, Henderson BE, Hoogstraal H, Johnson KM, Shelokov A. A review of Soviet viral hemorrhagic fever, 1969. J Infect Dis 1970;122:435-53.
4. Astakhova T, Slonova R, Tkachenko E, Bondarenko A, Kosoy M, Kushnarev E. Study of the role of hantavirus serotypes in the etiology of hemorrhagic fever with renal syndrome in the Far East of the USSR. Vopr Virusol 1990;35:492-4. [In Russian.]
5. Kosoy M, Slonova R, Mills J, Mandel E, Childs J. Community structure and prevalence of hantavirus infection in rodents: a geographic division of the enzootic area in Far Eastern Russia. J Vect Ecol 1997;22:52-63.
6. Horling J, Chizhikov V, Lundkvist A, Jonsson M, Ivanov L, Dekonenko A, et al. Khabarovsk virus: a phylogenetically and serologically distinct hantavirus isolated from *Microtus fortis* trapped in far-east Russia. J Gen Virol 1996;77:687-94.
7. Yashina L, Patrushev N, Ivanov L, Slonova R, Mishin V, Kompanez G, et al. Genetic diversity of hantaviruses associated with hemorrhagic fever with renal syndrome in the far east of Russia. Virus Res 2000;70:31-44.

### International Conference on Emerging Infectious Diseases, 2002

The National Center for Infectious Diseases, Centers for Disease Control and Prevention, has scheduled the third International Conference on Emerging Infectious Diseases (ICEID2002) for March 24-27, 2002, at the Hyatt Regency Hotel, Atlanta, Georgia, USA. More than 2,500 participants are expected, representing many nations and disciplines. They will discuss the latest information on many aspects of new and reemerging pathogens, such as West Nile virus and issues concerning bioterrorism.

Conferrence information is available at http://www.cdc.gov/iceid

The Call for Abstracts is available at http://www.asmusa.org/mtgscr/iceido2.htm

Contact person is Charles Schable, cas1@cdc.gov

### OPPORTUNITIES FOR PEER REVIEWERS

The editors of Emerging Infectious Diseases seek to increase the roster of reviewers for manuscripts submitted by authors all over the world for publication in the journal. If you are interested in reviewing articles on emerging infectious disease topics, please e-mail your name, address, qualifications or curriculum vitae, and areas of expertise to eideditor@cdc.gov

At Emerging Infectious Diseases, we always request reviewers' consent before sending manuscripts, limit review requests to three or four per year, and allow 2-4 weeks for completion of reviews. We consider reviewers invaluable in the process of selecting and publishing high-quality scientific articles and acknowledge their contributions in the journal once a year.

Even though it brings no financial compensation, participation in the peer-review process is not without rewards. Manuscript review provides scientists at all stages of their career opportunities for professional growth by familiarizing them with research trends and the latest work in the field of infectious diseases and by improving their own skills for presenting scientific information through constructive criticism of those of their peers. To view the spectrum of articles we publish, information for authors, and our extensive style guide, visit the journal web site at www.cdc.gov/eid .

For more information on participating in the peer-review process of Emerging Infectious Diseases, e-mail eideditor@cdc.gov or call the journal office at 404-371-5329.

